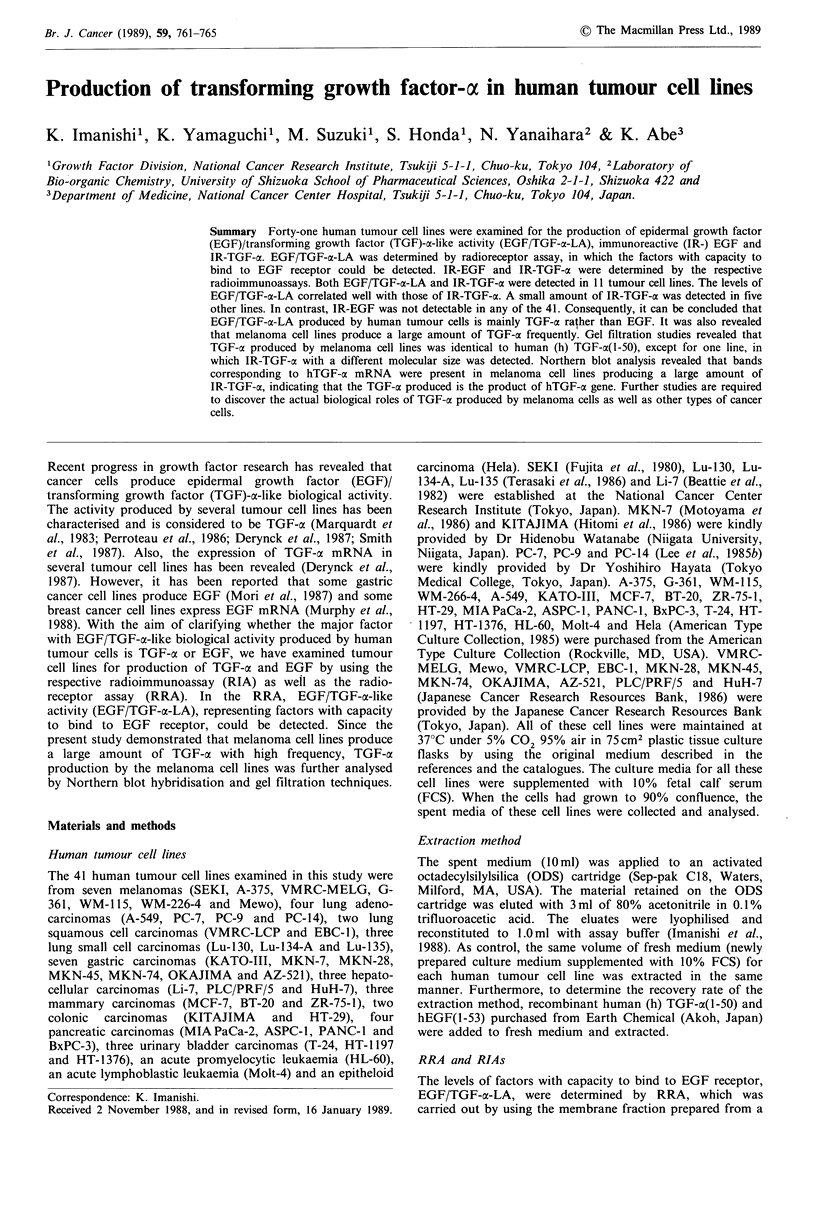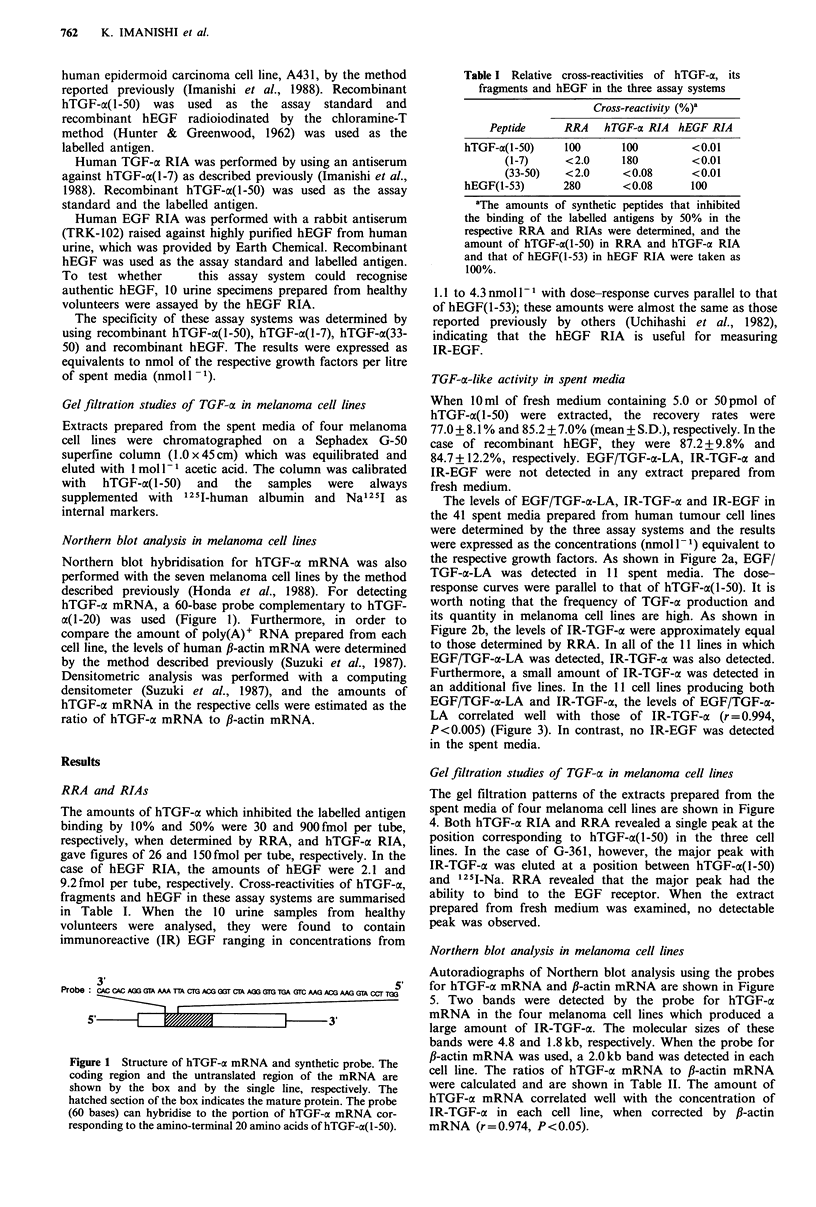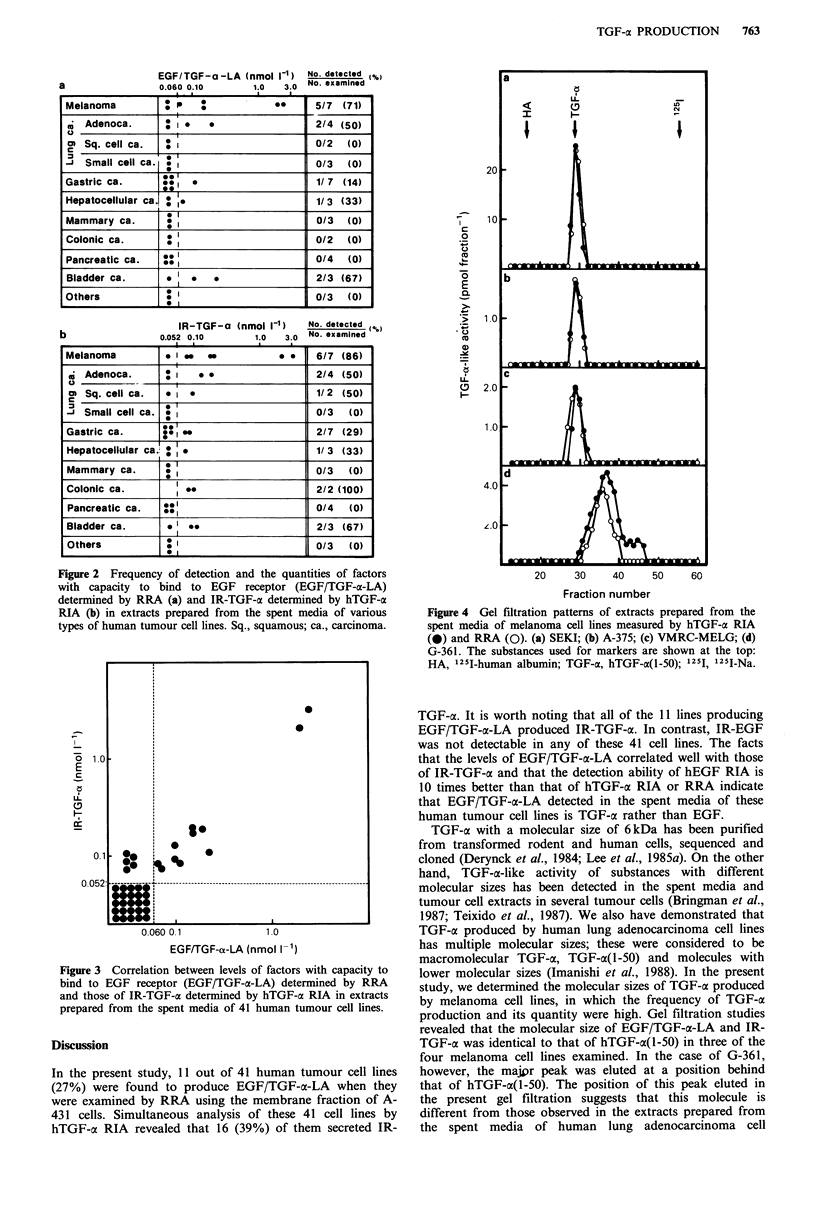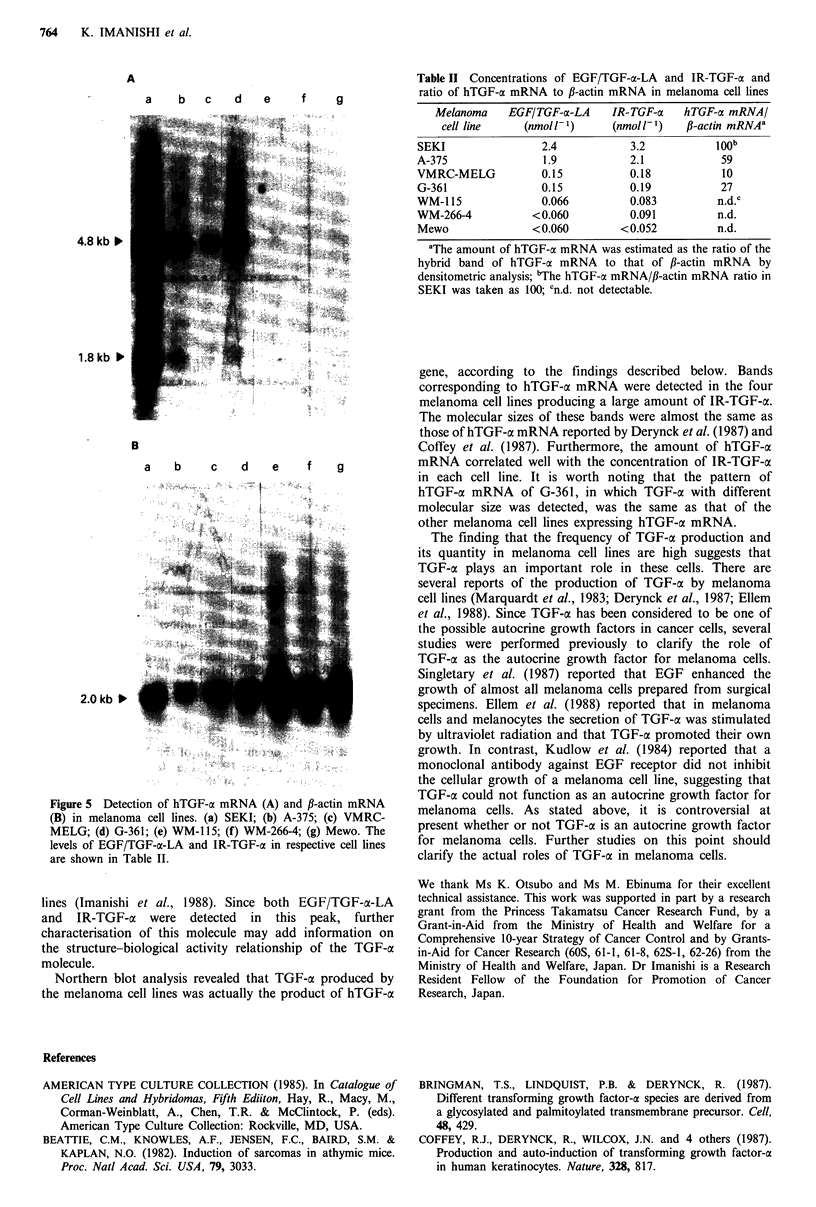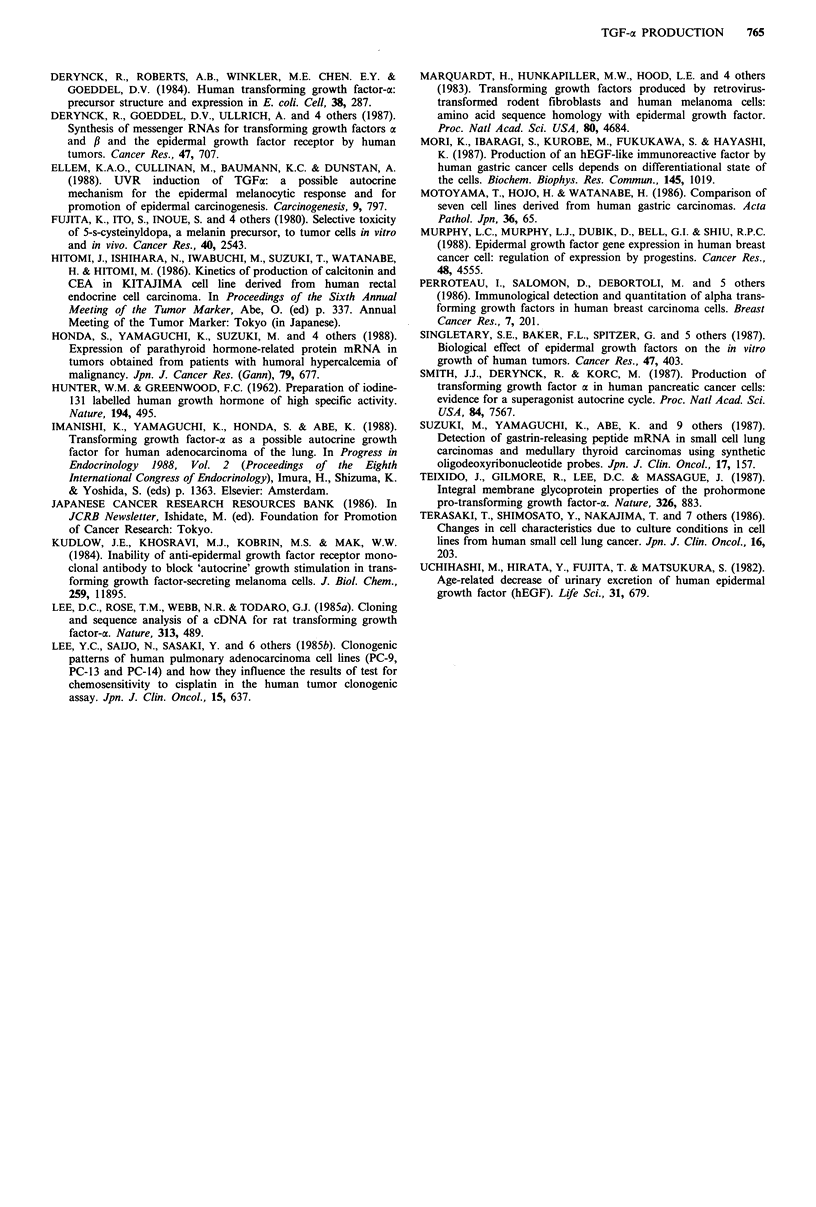# Production of transforming growth factor-alpha in human tumour cell lines.

**DOI:** 10.1038/bjc.1989.159

**Published:** 1989-05

**Authors:** K. Imanishi, K. Yamaguchi, M. Suzuki, S. Honda, N. Yanaihara, K. Abe

**Affiliations:** Growth Factor Division, National Cancer Research Institute, Tokyo, Japan.

## Abstract

**Images:**


*Br. J. Cancer* (1989), **59**, 761–765 © The Macmillan Press Ltd., 1989

### Production of transforming growth factor-α in human tumour cell lines

K. Imanishi[1], K. Yamaguchi[1], M. Suzuki[1], S. Honda[1], N. Yanaihara[2] & K. Abe[3]

[1]*Growth Factor Division, National Cancer Research Institute, Tsukiji 5-1-1, Chuo-ku, Tokyo 104,* [2]*Laboratory of Bio-organic Chemistry, University of Shizuoka School of Pharmaceutical Sciences, Oshika 2-1-1, Shizuoka 422 and* [3]*Department of Medicine, National Cancer Center Hospital, Tsukiji 5-1-1, Chuo-ku, Tokyo 104, Japan.*

**Summary** Forty-one human tumour cell lines were examined for the production of epidermal growth factor (EGF)/transforming growth factor (TGF)-α-like activity (EGF/TGF-α-LA), immunoreactive (IR-) EGF and IR-TGF-α. EGF/TGF-α-LA was determined by radioreceptor assay, in which the factors with capacity to bind to EGF receptor could be detected. IR-EGF and IR-TGF-α were determined by the respective radioimmunoassays. Both EGF/TGF-α-LA and IR-TGF-α were detected in 11 tumour cell lines. The levels of EGF/TGF-α-LA correlated well with those of IR-TGF-α. A small amount of IR-TGF-α was detected in five other lines. In contrast, IR-EGF was not detectable in any of the 41. Consequently, it can be concluded that EGF/TGF-α-LA produced by human tumour cells is mainly TGF-α rather than EGF. It was also revealed that melanoma cell lines produce a large amount of TGF-α frequently. Gel filtration studies revealed that TGF-α produced by melanoma cell lines was identical to human (h) TGF-α(1-50), except for one line, in which IR-TGF-α with a different molecular size was detected. Northern blot analysis revealed that bands corresponding to hTGF-α mRNA were present in melanoma cell lines producing a large amount of IR-TGF-α, indicating that the TGF-α produced is the product of hTGF-α gene. Further studies are required to discover the actual biological roles of TGF-α produced by melanoma cells as well as other types of cancer cells.

Recent progress in growth factor research has revealed that cancer cells produce epidermal growth factor (EGF)/ transforming growth factor (TGF)-α-like biological activity. The activity produced by several tumour cell lines has been characterised and is considered to be TGF-α (Marquardt *et al.*, 1983; Perroteau *et al.*, 1986; Derynck *et al.*, 1987; Smith *et al.*, 1987). Also, the expression of TGF-α mRNA in several tumour cell lines has been revealed (Derynck *et al.*, 1987). However, it has been reported that some gastric cancer cell lines produce EGF (Mori *et al.*, 1987) and some breast cancer cell lines express EGF mRNA (Murphy *et al.*, 1988). With the aim of clarifying whether the major factor with EGF/TGF-α-like biological activity produced by human tumour cells is TGF-α or EGF, we have examined tumour cell lines for production of TGF-α and EGF by using the respective radioimmunoassay (RIA) as well as the radioreceptor assay (RRA). In the RRA, EGF/TGF-α-like activity (EGF/TGF-α-LA), representing factors with capacity to bind to EGF receptor, could be detected. Since the present study demonstrated that melanoma cell lines produce a large amount of TGF-α with high frequency, TGF-α production by the melanoma cell lines was further analysed by Northern blot hybridisation and gel filtration techniques.

### Materials and methods

### *Human tumour cell lines*

The 41 human tumour cell lines examined in this study were from seven melanomas (SEKI, A-375, VMRC-MELG, G-361, WM-115, WM-226-4 and Mewo), four lung adenocarcinomas (A-549, PC-7, PC-9 and PC-14), two lung squamous cell carcinomas (VMRC-LCP and EBC-1), three lung small cell carcinomas (Lu-130, Lu-134-A and Lu-135), seven gastric carcinomas (KATO-III, MKN-7, MKN-28, MKN-45, MKN-74, OKAJIMA and AZ-521), three hepatocellular carcinomas (Li-7, PLC/PRF/5 and HuH-7), three mammary carcinomas (MCF-7, BT-20 and ZR-75-1), two colonic carcinomas (KITAJIMA and HT-29), four pancreatic carcinomas (MIA PaCa-2, ASPC-1, PANC-1 and BxPC-3), three urinary bladder carcinomas (T-24, HT-1197 and HT-1376), an acute promyelocytic leukaemia (HL-60), an acute lymphoblastic leukaemia (Molt-4) and an epitheloid carcinoma (Hela). SEKI (Fujita *et al.*, 1980), Lu-130, Lu-134-A, Lu-135 (Terasaki *et al.*, 1986) and Li-7 (Beattie *et al.*, 1982) were established at the National Cancer Center Research Institute (Tokyo, Japan). MKN-7 (Motoyama *et al.*, 1986) and KITAJIMA (Hitomi *et al.*, 1986) were kindly provided by Dr Hidenobu Watanabe (Niigata University, Niigata, Japan). PC-7, PC-9 and PC-14 (Lee *et al.*, 1985*b*) were kindly provided by Dr Yoshihiro Hayata (Tokyo Medical College, Tokyo, Japan). A-375, G-361, WM-115, WM-266-4, A-549, KATO-III, MCF-7, BT-20, ZR-75-1, HT-29, MIA PaCa-2, ASPC-1, PANC-1, BxPC-3, T-24, HT-1197, HT-1376, HL-60, Molt-4 and Hela (American Type Culture Collection, 1985) were purchased from the American Type Culture Collection (Rockville, MD, USA). VMRC-MELG, Mewo, VMRC-LCP, EBC-1, MKN-28, MKN-45, MKN-74, OKAJIMA, AZ-521, PLC/PRF/5 and HuH-7 (Japanese Cancer Research Resources Bank, 1986) were provided by the Japanese Cancer Research Resources Bank (Tokyo, Japan). All of these cell lines were maintained at 37°C under 5% $CO_2$ 95% air in 75 $cm^2$ plastic tissue culture flasks by using the original medium described in the references and the catalogues. The culture media for all these cell lines were supplemented with 10% fetal calf serum (FCS). When the cells had grown to 90% confluence, the spent media of these cell lines were collected and analysed.

### *Extraction method*

The spent medium (10 ml) was applied to an activated octadecylsilylsilica (ODS) cartridge (Sep-pak C18, Waters, Milford, MA, USA). The material retained on the ODS cartridge was eluted with 3 ml of 80% acetonitrile in 0.1% trifluoroacetic acid. The eluates were lyophilised and reconstituted to 1.0 ml with assay buffer (Imanishi *et al.*, 1988). As control, the same volume of fresh medium (newly prepared culture medium supplemented with 10% FCS) for each human tumour cell line was extracted in the same manner. Furthermore, to determine the recovery rate of the extraction method, recombinant human (h) TGF-α(1-50) and hEGF(1-53) purchased from Earth Chemical (Akoh, Japan) were added to fresh medium and extracted.

### *RRA and RIAs*

The levels of factors with capacity to bind to EGF receptor, EGF/TGF-α-LA, were determined by RRA, which was carried out by using the membrane fraction prepared from a

Correspondence: K. Imanishi.
Received 2 November 1988, and in revised form, 16 January 1989.

human epidermoid carcinoma cell line, A431, by the method reported previously (Imanishi *et al.*, 1988). Recombinant hTGF-α(1-50) was used as the assay standard and recombinant hEGF radioiodinated by the chloramine-T method (Hunter & Greenwood, 1962) was used as the labelled antigen.

Human TGF-α RIA was performed by using an antiserum against hTGF-α(1-7) as described previously (Imanishi *et al.*, 1988). Recombinant hTGF-α(1-50) was used as the assay standard and the labelled antigen.

Human EGF RIA was performed with a rabbit antiserum (TRK-102) raised against highly purified hEGF from human urine, which was provided by Earth Chemical. Recombinant hEGF was used as the assay standard and labelled antigen. To test whether this assay system could recognise authentic hEGF, 10 urine specimens prepared from healthy volunteers were assayed by the hEGF RIA.

The specificity of these assay systems was determined by using recombinant hTGF-α(1-50), hTGF-α(1-7), hTGF-α(33-50) and recombinant hEGF. The results were expressed as equivalents to nmol of the respective growth factors per litre of spent media ($nmol\,l^{-1}$).

*Gel filtration studies of TGF-α in melanoma cell lines*

Extracts prepared from the spent media of four melanoma cell lines were chromatographed on a Sephadex G-50 superfine column (1.0 × 45 cm) which was equilibrated and eluted with $1\,mol\,l^{-1}$ acetic acid. The column was calibrated with hTGF-α(1-50) and the samples were always supplemented with $^{125}$I-human albumin and $Na^{125}I$ as internal markers.

*Northern blot analysis in melanoma cell lines*

Northern blot hybridisation for hTGF-α mRNA was also performed with the seven melanoma cell lines by the method described previously (Honda *et al.*, 1988). For detecting hTGF-α mRNA, a 60-base probe complementary to hTGF-α(1-20) was used (Figure 1). Furthermore, in order to compare the amount of poly(A)$^+$ RNA prepared from each cell line, the levels of human β-actin mRNA were determined by the method described previously (Suzuki *et al.*, 1987). Densitometric analysis was performed with a computing densitometer (Suzuki *et al.*, 1987), and the amounts of hTGF-α mRNA in the respective cells were estimated as the ratio of hTGF-α mRNA to β-actin mRNA.

### Results

*RRA and RIAs*

The amounts of hTGF-α which inhibited the labelled antigen binding by 10% and 50% were 30 and 900 fmol per tube, respectively, when determined by RRA, and hTGF-α RIA, gave figures of 26 and 150 fmol per tube, respectively. In the case of hEGF RIA, the amounts of hEGF were 2.1 and 9.2 fmol per tube, respectively. Cross-reactivities of hTGF-α, fragments and hEGF in these assay systems are summarised in Table I. When the 10 urine samples from healthy volunteers were analysed, they were found to contain immunoreactive (IR) EGF ranging in concentrations from 1.1 to $4.3\,nmol\,l^{-1}$ with dose–response curves parallel to that of hEGF(1-53); these amounts were almost the same as those reported previously by others (Uchihashi *et al.*, 1982), indicating that the hEGF RIA is useful for measuring IR-EGF.

**Table I** Relative cross-reactivities of hTGF-α, its fragments and hEGF in the three assay systems

| | *Cross-reactivity* (%)[a] | | |
|---|---|---|---|
| *Peptide* | *RRA* | *hTGF-α RIA* | *hEGF RIA* |
| hTGF-α(1-50) | 100 | 100 | <0.01 |
| (1-7) | <2.0 | 180 | <0.01 |
| (33-50) | <2.0 | <0.08 | <0.01 |
| hEGF(1-53) | 280 | <0.08 | 100 |

[a]The amounts of synthetic peptides that inhibited the binding of the labelled antigens by 50% in the respective RRA and RIAs were determined, and the amount of hTGF-α(1-50) in RRA and hTGF-α RIA and that of hEGF(1-53) in hEGF RIA were taken as 100%.

**Figure 1** Structure of hTGF-α mRNA and synthetic probe. The coding region and the untranslated region of the mRNA are shown by the box and by the single line, respectively. The hatched section of the box indicates the mature protein. The probe (60 bases) can hybridise to the portion of hTGF-α mRNA corresponding to the amino-terminal 20 amino acids of hTGF-α(1-50).

*TGF-α-like activity in spent media*

When 10 ml of fresh medium containing 5.0 or 50 pmol of hTGF-α(1-50) were extracted, the recovery rates were 77.0 ± 8.1% and 85.2 ± 7.0% (mean ± S.D.), respectively. In the case of recombinant hEGF, they were 87.2 ± 9.8% and 84.7 ± 12.2%, respectively. EGF/TGF-α-LA, IR-TGF-α and IR-EGF were not detected in any extract prepared from fresh medium.

The levels of EGF/TGF-α-LA, IR-TGF-α and IR-EGF in the 41 spent media prepared from human tumour cell lines were determined by the three assay systems and the results were expressed as the concentrations ($nmol\,l^{-1}$) equivalent to the respective growth factors. As shown in Figure 2a, EGF/TGF-α-LA was detected in 11 spent media. The dose–response curves were parallel to that of hTGF-α(1-50). It is worth noting that the frequency of TGF-α production and its quantity in melanoma cell lines are high. As shown in Figure 2b, the levels of IR-TGF-α were approximately equal to those determined by RRA. In all of the 11 lines in which EGF/TGF-α-LA was detected, IR-TGF-α was also detected. Furthermore, a small amount of IR-TGF-α was detected in an additional five lines. In the 11 cell lines producing both EGF/TGF-α-LA and IR-TGF-α, the levels of EGF/TGF-α-LA correlated well with those of IR-TGF-α ($r = 0.994$, $P < 0.005$) (Figure 3). In contrast, no IR-EGF was detected in the spent media.

*Gel filtration studies of TGF-α in melanoma cell lines*

The gel filtration patterns of the extracts prepared from the spent media of four melanoma cell lines are shown in Figure 4. Both hTGF-α RIA and RRA revealed a single peak at the position corresponding to hTGF-α(1-50) in the three cell lines. In the case of G-361, however, the major peak with IR-TGF-α was eluted at a position between hTGF-α(1-50) and $^{125}$I-Na. RRA revealed that the major peak had the ability to bind to the EGF receptor. When the extract prepared from fresh medium was examined, no detectable peak was observed.

*Northern blot analysis in melanoma cell lines*

Autoradiographs of Northern blot analysis using the probes for hTGF-α mRNA and β-actin mRNA are shown in Figure 5. Two bands were detected by the probe for hTGF-α mRNA in the four melanoma cell lines which produced a large amount of IR-TGF-α. The molecular sizes of these bands were 4.8 and 1.8 kb, respectively. When the probe for β-actin mRNA was used, a 2.0 kb band was detected in each cell line. The ratios of hTGF-α mRNA to β-actin mRNA were calculated and are shown in Table II. The amount of hTGF-α mRNA correlated well with the concentration of IR-TGF-α in each cell line, when corrected by β-actin mRNA ($r = 0.974$, $P < 0.05$).

**Figure 2** Frequency of detection and the quantities of factors with capacity to bind to EGF receptor (EGF/TGF-α-LA) determined by RRA (**a**) and IR-TGF-α determined by hTGF-α RIA (**b**) in extracts prepared from the spent media of various types of human tumour cell lines. Sq., squamous; ca., carcinoma.

**Figure 3** Correlation between levels of factors with capacity to bind to EGF receptor (EGF/TGF-α-LA) determined by RRA and those of IR-TGF-α determined by hTGF-α RIA in extracts prepared from the spent media of 41 human tumour cell lines.

### Discussion

In the present study, 11 out of 41 human tumour cell lines (27%) were found to produce EGF/TGF-α-LA when they were examined by RRA using the membrane fraction of A-431 cells. Simultaneous analysis of these 41 cell lines by hTGF-α RIA revealed that 16 (39%) of them secreted IR-

**Figure 4** Gel filtration patterns of extracts prepared from the spent media of melanoma cell lines measured by hTGF-α RIA (●) and RRA (○). (**a**) SEKI; (**b**) A-375; (**c**) VMRC-MELG; (**d**) G-361. The substances used for markers are shown at the top: HA, $^{125}$I-human albumin; TGF-α, hTGF-α(1-50); $^{125}$I, $^{125}$I-Na.

TGF-α. It is worth noting that all of the 11 lines producing EGF/TGF-α-LA produced IR-TGF-α. In contrast, IR-EGF was not detectable in any of these 41 cell lines. The facts that the levels of EGF/TGF-α-LA correlated well with those of IR-TGF-α and that the detection ability of hEGF RIA is 10 times better than that of hTGF-α RIA or RRA indicate that EGF/TGF-α-LA detected in the spent media of these human tumour cell lines is TGF-α rather than EGF.

TGF-α with a molecular size of 6 kDa has been purified from transformed rodent and human cells, sequenced and cloned (Derynck *et al.*, 1984; Lee *et al.*, 1985*a*). On the other hand, TGF-α-like activity of substances with different molecular sizes has been detected in the spent media and tumour cell extracts in several tumour cells (Bringman *et al.*, 1987; Teixido *et al.*, 1987). We also have demonstrated that TGF-α produced by human lung adenocarcinoma cell lines has multiple molecular sizes; these were considered to be macromolecular TGF-α, TGF-α(1-50) and molecules with lower molecular sizes (Imanishi *et al.*, 1988). In the present study, we determined the molecular sizes of TGF-α produced by melanoma cell lines, in which the frequency of TGF-α production and its quantity were high. Gel filtration studies revealed that the molecular size of EGF/TGF-α-LA and IR-TGF-α was identical to that of hTGF-α(1-50) in three of the four melanoma cell lines examined. In the case of G-361, however, the major peak was eluted at a position behind that of hTGF-α(1-50). The position of this peak eluted in the present gel filtration suggests that this molecule is different from those observed in the extracts prepared from the spent media of human lung adenocarcinoma cell

**Figure 5** Detection of hTGF-α mRNA (**A**) and β-actin mRNA (**B**) in melanoma cell lines. (**a**) SEKI; (**b**) A-375; (**c**) VMRC-MELG; (**d**) G-361; (**e**) WM-115; (**f**) WM-266-4; (**g**) Mewo. The levels of EGF/TGF-α-LA and IR-TGF-α in respective cell lines are shown in Table II.

lines (Imanishi *et al.*, 1988). Since both EGF/TGF-α-LA and IR-TGF-α were detected in this peak, further characterisation of this molecule may add information on the structure–biological activity relationship of the TGF-α molecule.

Northern blot analysis revealed that TGF-α produced by the melanoma cell lines was actually the product of hTGF-α gene, according to the findings described below. Bands corresponding to hTGF-α mRNA were detected in the four melanoma cell lines producing a large amount of IR-TGF-α. The molecular sizes of these bands were almost the same as those of hTGF-α mRNA reported by Derynck *et al.* (1987) and Coffey *et al.* (1987). Furthermore, the amount of hTGF-α mRNA correlated well with the concentration of IR-TGF-α in each cell line. It is worth noting that the pattern of hTGF-α mRNA of G-361, in which TGF-α with different molecular size was detected, was the same as that of the other melanoma cell lines expressing hTGF-α mRNA.

**Table II** Concentrations of EGF/TGF-α-LA and IR-TGF-α and ratio of hTGF-α mRNA to β-actin mRNA in melanoma cell lines

| *Melanoma cell line* | *EGF/TGF-α-LA* ($nmol\,l^{-1}$) | *IR-TGF-α* ($nmol\,l^{-1}$) | *hTGF-α mRNA/ β-actin mRNA*[a] |
|---|---|---|---|
| SEKI | 2.4 | 3.2 | 100[b] |
| A-375 | 1.9 | 2.1 | 59 |
| VMRC-MELG | 0.15 | 0.18 | 10 |
| G-361 | 0.15 | 0.19 | 27 |
| WM-115 | 0.066 | 0.083 | n.d.[c] |
| WM-266-4 | <0.060 | 0.091 | n.d. |
| Mewo | <0.060 | <0.052 | n.d. |

[a]The amount of hTGF-α mRNA was estimated as the ratio of the hybrid band of hTGF-α mRNA to that of β-actin mRNA by densitometric analysis; [b]The hTGF-α mRNA/β-actin mRNA ratio in SEKI was taken as 100; [c]n.d. not detectable.

The finding that the frequency of TGF-α production and its quantity in melanoma cell lines are high suggests that TGF-α plays an important role in these cells. There are several reports of the production of TGF-α by melanoma cell lines (Marquardt *et al.*, 1983; Derynck *et al.*, 1987; Ellem *et al.*, 1988). Since TGF-α has been considered to be one of the possible autocrine growth factors in cancer cells, several studies were performed previously to clarify the role of TGF-α as the autocrine growth factor for melanoma cells. Singletary *et al.* (1987) reported that EGF enhanced the growth of almost all melanoma cells prepared from surgical specimens. Ellem *et al.* (1988) reported that in melanoma cells and melanocytes the secretion of TGF-α was stimulated by ultraviolet radiation and that TGF-α promoted their own growth. In contrast, Kudlow *et al.* (1984) reported that a monoclonal antibody against EGF receptor did not inhibit the cellular growth of a melanoma cell line, suggesting that TGF-α could not function as an autocrine growth factor for melanoma cells. As stated above, it is controversial at present whether or not TGF-α is an autocrine growth factor for melanoma cells. Further studies on this point should clarify the actual roles of TGF-α in melanoma cells.

We thank Ms K. Otsubo and Ms M. Ebinuma for their excellent technical assistance. This work was supported in part by a research grant from the Princess Takamatsu Cancer Research Fund, by a Grant-in-Aid from the Ministry of Health and Welfare for a Comprehensive 10-year Strategy of Cancer Control and by Grants-in-Aid for Cancer Research (60S, 61-1, 61-8, 62S-1, 62-26) from the Ministry of Health and Welfare, Japan. Dr Imanishi is a Research Resident Fellow of the Foundation for Promotion of Cancer Research, Japan.

### References

AMERICAN TYPE CULTURE COLLECTION (1985). In *Catalogue of Cell Lines and Hybridomas, Fifth Ediiton*, Hay, R., Macy, M., Corman-Weinblatt, A., Chen, T.R. & McClintock, P. (eds). American Type Culture Collection: Rockville, MD, USA.

BEATTIE, C.M., KNOWLES, A.F., JENSEN, F.C., BAIRD, S.M. & KAPLAN, N.O. (1982). Induction of sarcomas in athymic mice. *Proc. Natl Acad. Sci. USA*, **79**, 3033.

BRINGMAN, T.S., LINDQUIST, P.B. & DERYNCK, R. (1987). Different transforming growth factor-α species are derived from a glycosylated and palmitoylated transmembrane precursor. *Cell*, **48**, 429.

COFFEY, R.J., DERYNCK, R., WILCOX, J.N. and 4 others (1987). Production and auto-induction of transforming growth factor-α in human keratinocytes. *Nature*, **328**, 817.

DERYNCK, R., ROBERTS, A.B., WINKLER, M.E. CHEN. E.Y. & GOEDDEL, D.V. (1984). Human transforming growth factor-α: precursor structure and expression in *E. coli*. *Cell*, **38**, 287.

DERYNCK, R., GOEDDEL, D.V., ULLRICH, A. and 4 others (1987). Synthesis of messenger RNAs for transforming growth factors α and β and the epidermal growth factor receptor by human tumors. *Cancer Res.*, **47**, 707.

ELLEM, K.A.O., CULLINAN, M., BAUMANN, K.C. & DUNSTAN, A. (1988). UVR induction of TGFα: a possible autocrine mechanism for the epidermal melanocytic response and for promotion of epidermal carcinogenesis. *Carcinogenesis*, **9**, 797.

FUJITA, K., ITO, S., INOUE, S. and 4 others (1980). Selective toxicity of 5-s-cysteinyldopa, a melanin precursor, to tumor cells *in vitro* and *in vivo*. *Cancer Res.*, **40**, 2543.

HITOMI, J., ISHIHARA, N., IWABUCHI, M., SUZUKI, T., WATANABE, H. & HITOMI, M. (1986). Kinetics of production of calcitonin and CEA in KITAJIMA cell line derived from human rectal endocrine cell carcinoma. In *Proceedings of the Sixth Annual Meeting of the Tumor Marker*, Abe, O. (ed) p. 337. Annual Meeting of the Tumor Marker: Tokyo (in Japanese).

HONDA, S., YAMAGUCHI, K., SUZUKI, M. and 4 others (1988). Expression of parathyroid hormone-related protein mRNA in tumors obtained from patients with humoral hypercalcemia of malignancy. *Jpn. J. Cancer Res.* (*Gann*), **79**, 677.

HUNTER, W.M. & GREENWOOD, F.C. (1962). Preparation of iodine-131 labelled human growth hormone of high specific activity. *Nature*, **194**, 495.

IMANISHI, K., YAMAGUCHI, K., HONDA, S. & ABE, K. (1988). Transforming growth factor-α as a possible autocrine growth factor for human adenocarcinoma of the lung. In *Progress in Endocrinology 1988, Vol. 2* (*Proceedings of the Eighth International Congress of Endocrinology*), Imura, H., Shizuma, K. & Yoshida, S. (eds) p. 1363. Elsevier: Amsterdam.

JAPANESE CANCER RESEARCH RESOURCES BANK (1986). In *JCRB Newsletter*, Ishidate, M. (ed). Foundation for Promotion of Cancer Research: Tokyo.

KUDLOW, J.E., KHOSRAVI, M.J., KOBRIN, M.S. & MAK, W.W. (1984). Inability of anti-epidermal growth factor receptor monoclonal antibody to block 'autocrine' growth stimulation in transforming growth factor-secreting melanoma cells. *J. Biol. Chem.*, **259**, 11895.

LEE, D.C., ROSE, T.M., WEBB, N.R. & TODARO, G.J. (1985*a*). Cloning and sequence analysis of a cDNA for rat transforming growth factor-α. *Nature*, **313**, 489.

LEE, Y.C., SAIJO, N., SASAKI, Y. and 6 others (1985*b*). Clonogenic patterns of human pulmonary adenocarcinoma cell lines (PC-9, PC-13 and PC-14) and how they influence the results of test for chemosensitivity to cisplatin in the human tumor clonogenic assay. *Jpn. J. Clin. Oncol.*, **15**, 637.

MARQUARDT, H., HUNKAPILLER, M.W., HOOD, L.E. and 4 others (1983). Transforming growth factors produced by retrovirus-transformed rodent fibroblasts and human melanoma cells: amino acid sequence homology with epidermal growth factor. *Proc. Natl Acad. Sci. USA*, **80**, 4684.

MORI, K., IBARAGI, S., KUROBE, M., FUKUKAWA, S. & HAYASHI, K. (1987). Production of an hEGF-like immunoreactive factor by human gastric cancer cells depends on differentiational state of the cells. *Biochem. Biophys. Res. Commun.*, **145**, 1019.

MOTOYAMA, T., HOJO, H. & WATANABE, H. (1986). Comparison of seven cell lines derived from human gastric carcinomas. *Acta Pathol. Jpn*, **36**, 65.

MURPHY, L.C., MURPHY, L.J., DUBIK, D., BELL, G.I. & SHIU, R.P.C. (1988). Epidermal growth factor gene expression in human breast cancer cell: regulation of expression by progestins. *Cancer Res.*, **48**, 4555.

PERROTEAU, I., SALOMON, D., DEBORTOLI, M. and 5 others (1986). Immunological detection and quantitation of alpha transforming growth factors in human breast carcinoma cells. *Breast Cancer Res.*, **7**, 201.

SINGLETARY, S.E., BAKER, F.L., SPITZER, G. and 5 others (1987). Biological effect of epidermal growth factors on the *in vitro* growth of human tumors. *Cancer Res.*, **47**, 403.

SMITH, J.J., DERYNCK, R. & KORC, M. (1987). Production of transforming growth factor α in human pancreatic cancer cells: evidence for a superagonist autocrine cycle. *Proc. Natl Acad. Sci. USA*, **84**, 7567.

SUZUKI, M., YAMAGUCHI, K., ABE, K. and 9 others (1987). Detection of gastrin-releasing peptide mRNA in small cell lung carcinomas and medullary thyroid carcinomas using synthetic oligodeoxyribonucleotide probes. *Jpn. J. Clin. Oncol.*, **17**, 157.

TEIXIDO, J., GILMORE, R., LEE, D.C. & MASSAGUE, J. (1987). Integral membrane glycoprotein properties of the prohormone pro-transforming growth factor-α. *Nature*, **326**, 883.

TERASAKI, T., SHIMOSATO, Y., NAKAJIMA, T. and 7 others (1986). Changes in cell characteristics due to culture conditions in cell lines from human small cell lung cancer. *Jpn. J. Clin. Oncol.*, **16**, 203.

UCHIHASHI, M., HIRATA, Y., FUJITA, T. & MATSUKURA, S. (1982). Age-related decrease of urinary excretion of human epidermal growth factor (hEGF). *Life Sci.*, **31**, 679.